# A comparison of the upright seated versus left lateral decubitus position during pediatric sedation‐free transnasal endoscopy

**DOI:** 10.1002/jpr3.70248

**Published:** 2026-10-06

**Authors:** Catherine Nguyen, Molly Mackensen, Sophie Branen, Katherine Vaidy, Rose Lee

**Affiliations:** ^1^ Medical College of Wisconsin Milwaukee Wisconsin USA; ^2^ Children's Hospital of Wisconsin Milwaukee Wisconsin USA; ^3^ Department of Pediatrics, Division of Pediatric Gastroenterology, Hepatology, and Nutrition Medical College of Wisconsin Milwaukee Wisconsin USA

**Keywords:** children, dysphagia, eosinophilic esophagitis, esophagoscopy, unsedated endoscopy

## Abstract

**Objectives:**

Although sedation‐free transnasal endoscopy (TNE) is conventionally performed in an upright seated position in pediatric patients, emerging data suggest that the left lateral decubitus (LLD) position may improve both endoscopic visualization and patient comfort and safety. This study compared TNE outcomes and tolerance in pediatric patients using the upright versus LLD position.

**Methods:**

A retrospective review was conducted on 110 pediatric patients who underwent sedation‐free TNE from September 2024 to November 2025. Three cases were excluded for scope failure during the procedure. Data including patient demographics, procedure time, TNEase (patient tolerance) score, and adverse events were collected.

**Results:**

Of the 107 cases, 71 were performed upright, 31 LLD, and 5 converted from upright to LLD due to syncope. Four cases of emesis and two cases of minor epistaxis occurred during upright TNE, while no major adverse events occurred during LLD TNE. There were no significant differences between upright and LLD TNE in patient age (upright 13.8 ± 2.8 vs. LLD 12.5 ± 3.3 years), mean TNEase score (1.1 vs. 1.4), or procedure times. For TNE‐esophagoscopy, mean procedure times were 5.1 (*n* = 55) and 5.5 min (*n* = 27) in the upright versus LLD position, respectively. Times were 6.1 (*n* = 12) versus 6.8 min (*n* = 4) for TNE‐esophagogastroscopy, and 17.7 (*n* = 4) versus 17.4 min (*n* = 5) for TNE‐esophagogastroduodenoscopy.

**Conclusions:**

The LLD position is a safe and effective alternative to upright TNE and is better tolerated in patients with orthostatic intolerance.

## Abstract (highlights)

**What is Known:**

- Sedation‐free transnasal endoscopy (TNE) is a safe and effective procedure for upper gastrointestinal evaluation and surveillance.
- The upright seated and left lateral decubitus (LLD) positions are both used in adult TNE, while the upright position is almost exclusively employed in pediatric TNE.
- A new study demonstrated that the LLD position in pediatric TNE is feasible and well‐tolerated.

**What is New:**

- The LLD position is clinically equivalent to the upright seated position in pediatric TNE.
- The standardization of both approaches in pediatric TNE may help to expand the use of TNE across diverse clinical sites.

## 1 INTRODUCTION

Sedation‐free transnasal endoscopy (TNE) is a reliable, minimally invasive alternative to sedated esophagogastroduodenoscopy (EGD) for evaluation of the upper gastrointestinal (GI) tract.^1^ While EGD remains the gold standard for diagnosing and monitoring upper GI disorders, the use of general anesthesia or sedation during EGD poses various risks, particularly in patients with pre‐existing health conditions or pediatric patients requiring serial endoscopic evaluation.^2^, ^3^ Potential complications of frequent and/or prolonged sedation include hemodynamic instability, impaired cardiopulmonary function, and neurotoxicity in the developing brain.^3^, ^4^ TNE eliminates the risk of sedation‐associated complications and provides additional benefits such as an improved safety profile, high degree of patient tolerance, reduced cost, and shorter procedure time.^5^, ^6^, ^7^, ^8^ Importantly, biopsy specimens gathered during TNE are sufficient for diagnostic determination and are equivalent in histologic quality to those collected during conventional EGD.^7^

Originally devised by adult gastroenterologists in the 1990s, TNE involves passing an ultra‐thin endoscope through the nasal passageway using topical anesthetic to visualize the esophagus, stomach, and duodenum.^9^ Its current indications are broad, overlapping with many pathologies historically managed via EGD like dysphagia, reflux, eosinophilic esophagitis (EoE), Barrett's esophagus, vomiting, abdominal pain, and celiac disease.^10^ In adults, TNE is performed in either an upright seated or left lateral decubitus (LLD) position, whereas pediatric patients commonly undergo the procedure in the upright position.^11^ The rationale for this difference in procedural technique between adult and pediatric populations is not clearly defined. As such, there is growing interest in integrating the LLD position into clinical practice alongside the established upright position for pediatric TNE. In our previous pilot study of 13 pediatric cases, we demonstrated that TNE in the LLD position is feasible, well‐tolerated, and potentially preferrable to the upright seated position in patients with orthostatic intolerance.^12^ The aim of this current study was to build upon our initial findings by directly comparing TNE outcomes and tolerance levels between the upright seated versus LLD position in an expanded pediatric cohort.

## 2 METHODS

### 2.1 Ethics statement

This study was exempt from Institutional Review Board (IRB) review.

### 2.2 Study design and patient population

A retrospective analysis of pediatric patients who completed sedation‐free TNE from September 2024 to November 2025 at Children's Wisconsin hospital was conducted using data from electronic health records (EPIC). This cohort encompassed the 13 LLD patients previously reported in our pilot study, whose procedures were performed from September 2024 to February 2025.^12^

### 2.3 Procedural protocol and data collection

Endoscopies were performed in an outpatient procedure suite by two trained pediatric gastroenterologists. Sterile, single‐use 85 cm or 110 cm gastroscopes with a 3.5 mm outer diameter and 2 mm working channel were utilized. Prior to beginning, all patients were asked to select their preferred position for TNE: upright seated or LLD (Figure 1). Preprocedural treatment included oxymetazoline to reduce nasal congestion and 4% lidocaine spray in the nose and posterior oropharynx to minimize discomfort. Sweet items like candy were given to mitigate the bitterness of the topical anesthetic. Patients also wore virtual reality (VR) goggles throughout the procedure as a distraction technique to further decrease patient pain and anxiety.

**Figure 1:**
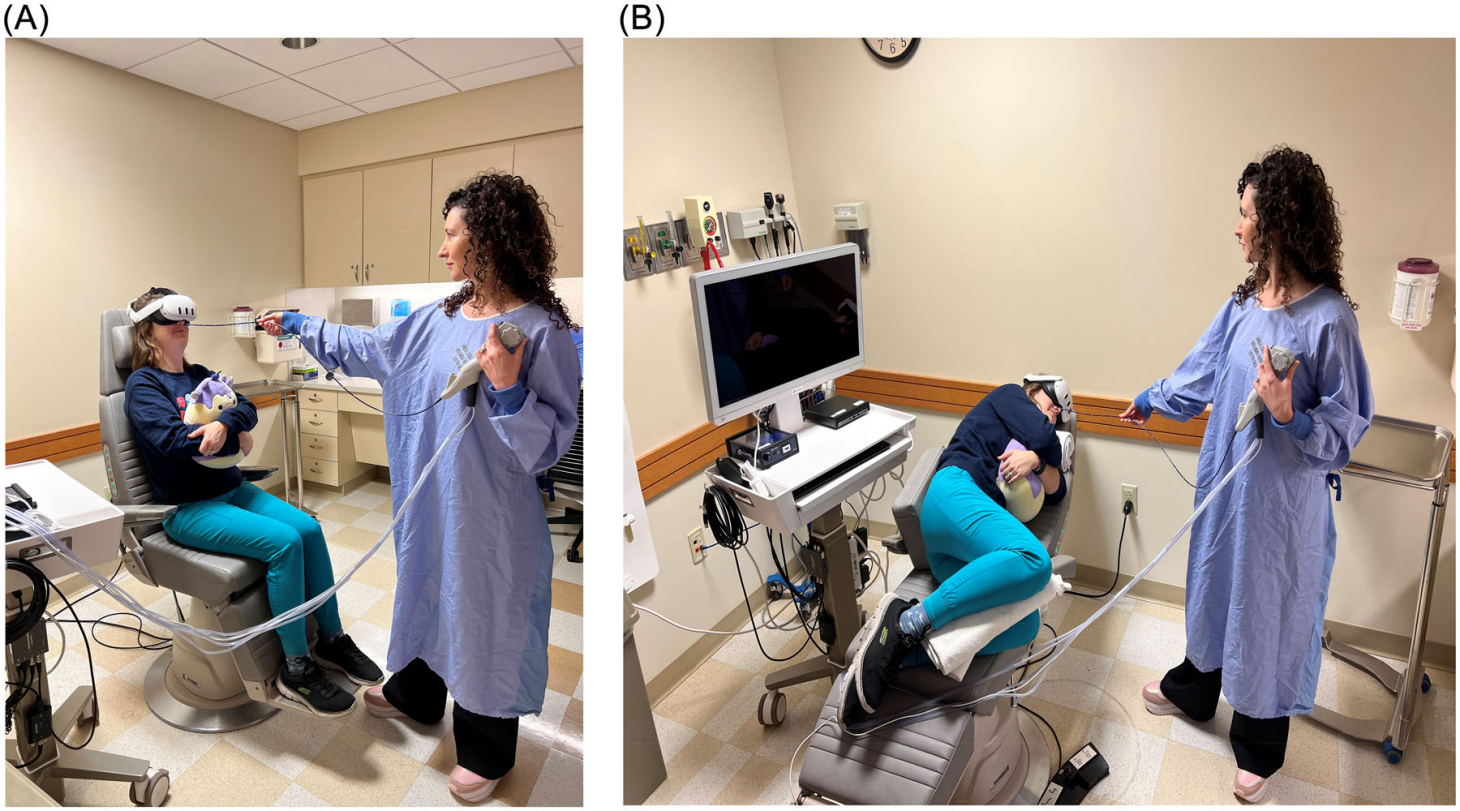
(A) Transnasal endoscopy in the upright seated position. (B) Transnasal endoscopy in the left lateral decubitus position.

Following the conclusion of the procedure, each TNE case was assigned a TNEase score by the presiding gastroenterologist, which serves as an indication of patient tolerance. Score values are defined as follows: 1 = performed on patient with ease, 2 = performed with minimal complaints, 3 = performed with moderate complaints, 4 = performed although with significant complaints and resistance, 5 = patient unable to tolerate and procedure was terminated.^13^ Study data including patient demographics, TNE indication, procedure type, procedure length, TNEase score, and adverse events were collected and analyzed.

### 2.4 Statistical methods

Descriptive statistics including means, standard deviations, frequencies, and percentages were used to summarize the data. All analyses were performed using Microsoft Excel Version 16.105.2.

## 3 RESULTS

A total of 110 patients ranging from ages 6 to 18 underwent pediatric, sedation‐free TNE from September 2024 to November 2025. Three cases were excluded from the study due to equipment malfunctioning during the procedure. TNE evaluation was conducted via esophagoscopy (TNE‐ESO) (*n* = 82), esophagogastroscopy (TNE‐EG) (*n* = 16), or esophagogastroduodenoscopy (TNE‐EGD) (*n* = 9). Primary indications for TNE included EoE (*n* = 91), dysphagia (*n* = 8), celiac disease (*n* = 4), reflux (*n* = 2), poor weight gain (*n* = 1), and vomiting (*n* = 1).

Seventy‐one procedures were completed in the upright seated position and thirty‐six in the LLD position, five of which were initiated upright but were converted to and reattempted in the LLD position due to syncopal episodes. These five patients successfully completed TNE in the LLD position without recurrent syncope and thus were included in the LLD TNE cohort (*n* = 36). There were four episodes of emesis and two instances of epistaxis during upright TNE. No major adverse events occurred during LLD TNE. A comparison of the study outcomes between the upright seated versus LLD cohorts is summarized in Table 1. Patients undergoing upright TNE had an average age of 13.8 years (SD = 2.8) compared to 12.5 years (SD = 3.3) in those undergoing LLD TNE. Mean TNEase scores did not significantly differ between groups (upright 1.1 ± 0.4 vs. LLD 1.4 ± 0.6). In both groups, TNEase scores did not exceed a value of 3 (performed with moderate complaints). Procedure lengths were also comparable: for TNE‐ESO, upright versus LLD mean procedure times were 5.1 (*n* = 55) versus 5.5 min (*n* = 27), respectively; for TNE‐EG, times were 6.1 (*n* = 12) versus 6.8 min (*n* = 4); for TNE‐EGD, times were 17.7 (*n* = 4) versus 17.4 min (*n* = 5).

**Table 1:** Comparison of patient demographics and study findings between upright seated and left lateral decubitus groups.

	Upright seated (*n* = 71)	Left lateral decubitus (*n* = 36)
Mean (SD) age (years)	13.8 (2.8)	12.5 (3.3)
Gender *n* (%)		
Male	61 (86%)	22 (61%)
Female	10 (14%)	14 (39%)
Mean (SD) TNEase score	1.1 (0.4)	1.4 (0.6)
TNEase score *n* (%)		
Score of 1	64 (90%)	25 (69%)
Score of 2	5 (7%)	9 (25%)
Score of 3	2 (3%)	2 (6%)
Score of 4	0 (0%)	0 (0%)
Score of 5	0 (0%)	0 (0%)
Mean (SD) TNE procedure length (min)		
Esophagoscopy	5.1 (1.6)	5.5 (2.2)
Esophagogastroscopy	6.1 (1.8)	6.8 (3.6)
Esophagogastroduodenoscopy	17.7 (7.4)	17.4 (4.7)

Abbreviations: SD, standard deviation; TNE, transnasal endoscopy.

## 4 DISCUSSION

Sedation‐free TNE is an emerging clinical tool for upper GI evaluation in pediatric patients. Although pediatric TNE is traditionally performed in the upright seated position, our previous study by Lee et al. demonstrated that TNE in the LLD position is safe and well‐tolerated in children.^12^ In this present study, we determined that the LLD position is a viable alternative to the standard upright position during pediatric TNE, as both positions yielded similar outcomes with regard to procedure duration and patient tolerance.

One difference to note between the two cohorts is the absence of major adverse events during LLD TNE, which suggests that LLD may reduce the likelihood of common TNE complications like vomiting, epistaxis, throat pain, or persistent nasal discomfort. Furthermore, five patients who experienced syncope while sitting upright successfully completed TNE without complication upon LLD repositioning. All five cases received a TNEase score of 1 (performed with ease) or 2 (performed with minimal complaint). These findings support observations made in our pilot study that LLD may be the preferred position in patients with orthostatic intolerance.^12^ Physiologically, the upright position poses a greater orthostatic challenge given increased venous pooling in the lower extremities due to gravity. By placing the patient in a horizontal and neutral plane, the LLD position improves venous return to the heart, reducing the risk of orthostatic hypotension and syncope.

Beyond these physiologic benefits, use of the LLD position can be implemented in diverse clinical environments. Traditional upright TNE requires a large, adjustable chair—often an otolaryngology or dental chair—to support the seated patient. This may not only be uncomfortable for smaller patients but also be impractical for medical centers with limited access to specialized equipment. By utilizing standard hospital beds or stretchers, the LLD position lowers institutional barriers to TNE program adoption and, importantly, enhances patient access to care. Additionally, the LLD orientation parallels the approach used in conventional EGD and replicates procedural ergonomics with which gastroenterologists are technically familiar. This can expedite the learning process for trainees, as well as facilitate greater implementation of TNE into clinical practice.

The main limitation of this study is its single‐center, retrospective design. Multi‐center, prospective studies are warranted to improve the generalizability of our findings and to control for potential confounding variables. Future research should strive to identify specific pediatric populations, aside from patients with a history of orthostatic intolerance, that would benefit most from the LLD approach.

## 5 CONCLUSION

To summarize, the LLD position is a safe and clinically equivalent alternative to the upright seated position in pediatric, sedation‐free TNE. By offering an additional validated approach, clinicians can provide patient‐centered care while maintaining a high degree of procedural integrity and efficacy.

## CONFLICT OF INTEREST STATEMENT

Rose Lee and Katherine Vaidy serve as consultants for EvoEndo Inc. The remaining authors declare no conflicts of interest.

## Data Availability

The data that support the findings of this study are available on request from the corresponding author. The data are not publicly available due to privacy or ethical restrictions.
